# Water Stress Identification of Winter Wheat Crop with State-of-the-Art AI Techniques and High-Resolution Thermal-RGB Imagery

**DOI:** 10.3390/plants11233344

**Published:** 2022-12-02

**Authors:** Narendra S. Chandel, Yogesh A. Rajwade, Kumkum Dubey, Abhilash K. Chandel, A. Subeesh, Mukesh K. Tiwari

**Affiliations:** 1 Agricultural Mechanization Division, ICAR—Central Institute of Agricultural Engineering, Bhopal 462038, MP, India; 2 Irrigation and Drainage Engineering Division, ICAR—Central Institute of Agricultural Engineering, Bhopal 462038, MP, India; 3 Department of Biological Systems Engineering, Virginia Tech Tidewater AREC, Suffolk, VA 23437, USA; 4 Center for Advanced Innovation in Agriculture (CAIA), Virginia Tech, Blacksburg, VA 24061, USA; 5 College of Agricultural Engineering and Technology, Anand Agricultural University, Godhra 389001, GJ, India

**Keywords:** winter wheat, crop water stress, canopy temperature, computer vision, irrigation management

## Abstract

Timely crop water stress detection can help precision irrigation management and minimize yield loss. A two-year study was conducted on non-invasive winter wheat water stress monitoring using state-of-the-art computer vision and thermal-RGB imagery inputs. Field treatment plots were irrigated using two irrigation systems (flood and sprinkler) at four rates (100, 75, 50, and 25% of crop evapotranspiration [ET_c_]). A total of 3200 images under different treatments were captured at critical growth stages, that is, 20, 35, 70, 95, and 108 days after sowing using a custom-developed thermal-RGB imaging system. Crop and soil response measurements of canopy temperature (T_c_), relative water content (RWC), soil moisture content (SMC), and relative humidity (RH) were significantly affected by the irrigation treatments showing the lowest T_c_ (22.5 ± 2 °C), and highest RWC (90%) and SMC (25.7 ± 2.2%) for 100% ET_c_, and highest T_c_ (28 ± 3 °C), and lowest RWC (74%) and SMC (20.5 ± 3.1%) for 25% ET_c_. The RGB and thermal imagery were then used as inputs to feature-extraction-based deep learning models (AlexNet, GoogLeNet, Inception V3, MobileNet V2, ResNet50) while, RWC, SMC, T_c_, and RH were the inputs to function-approximation models (Artificial Neural Network (ANN), Kernel Nearest Neighbor (KNN), Logistic Regression (LR), Support Vector Machine (SVM) and Long Short-Term Memory (DL-LSTM)) to classify stressed/non-stressed crops. Among the feature extraction-based models, ResNet50 outperformed other models showing a discriminant accuracy of 96.9% with RGB and 98.4% with thermal imagery inputs. Overall, classification accuracy was higher for thermal imagery compared to RGB imagery inputs. The DL-LSTM had the highest discriminant accuracy of 96.7% and less error among the function approximation-based models for classifying stress/non-stress. The study suggests that computer vision coupled with thermal-RGB imagery can be instrumental in high-throughput mitigation and management of crop water stress.

## 1. Introduction

Water stress forces leaf stomata closure, which reduces transpiration and increases canopy temperature (T_c_) [1]. Timely estimation of those stressors may not only help precision irrigation management but also minimize yield losses [2]. The penalty gap between actual and potential yield will widen further as a result of climate change that projects a decline in rainfall frequency, and rising ambient temperatures [3]. Water stress is typically assessed using xylem water potentials [4], canopy thermometry [5], and stomatal conductance measurements [6]. However, these methods are often invasive and tend to have limited sampling accuracy due to low throughput or point data acquisitions [7]. Non-invasive proximal or remote sensing techniques have emerged as high throughput alternatives for monitoring crop water stress through color features, reflectance, and thermal emissivity of the vegetable, fruit, and specialty crops [8,9,10]. However, monitoring crop water content using visible-range RGB imaging not only requires specific leaf orientation relative to the camera but also pre-defined illumination conditions. This limits the applicability of RGB imaging to determine water content in field conditions. Using scanner-type imaging devices could be a cost and time-effective alternative [11]. Unlike thermometry, T_c_ from thermal infrared imagery reflects upon the entire canopy emissivity profile, which is directly proportional to the canopy water content [9]. Thermal imagery (8000–14,000 nm) also outperforms color RGB imagery (400–700 nm), and reflectance characteristics in terms of robustness to characterize crop water stress [8,9]. Nonetheless, the adaptability of thermal imaging in agricultural production management is still at a nascent stage and is consistently evolving to maintain imaging quality against drastic variations in relative humidity and wind speeds. Thermal imaging cameras are also relatively more expensive than simple-to-operate RGB cameras. It is for these reasons; thermal imaging is still limitedly adopted as a golden standard for crop stress mapping. Above all, longwave infrared wavelengths (thermal imaging) have a higher penetrating capability over visible-range wavelengths, making them more reliable and sensitive to crop water content variations. Thermal imaging is therefore a better alternative for precision irrigation management unlike RGB imaging or using standard crop coefficients coupled with reference evapotranspiration [8,9].

RGB imagery has been used to assess crop water stress using different deep learning (DL) and machine learning (ML) techniques [10,12,13]. ML techniques derive unique features from input and output datasets, which could be used for discrimination between different object types or classes. ML techniques such as Naïve Bayes, artificial neural networks (ANNs), support vector machine (SVM), and random forests (RFs) have been widely used with RGB images for weed detection, biotic and abiotic stress identification and/or classification, yield predictions, and other crop phenotyping applications [14,15]. Thermal imagery has also been used with RFs and decision trees for crop water status monitoring in the vineyard and automated irrigation scheduling [16]. However, there are several limitations associated with ML techniques. The output quality is highly dependent on input data quality, the presence of noise and outliers, and other unaccounted biases that have been reported to significantly affect the model performance. Furthermore, ML techniques also require skilled operators [17] for defining input features that may also often affect the model performances through unintentional subjectivity and bias [10,12].

DL has emerged as an advanced vision-based learning technique that enables automated feature extraction without human dependencies unlike ML [18]. Pertinent to agricultural applications, crop phenological stages have been detected using a deep convolution neural network (DCNN) trained on RGB imagery [19,20]. Similarly, different DL techniques (AlexNet, GoogLeNet, and Inception V3) have also been used to classify non-stressed and water-stressed soybean, maize, and okra crops with digital RGB images [10]. Long Short Term Memory (LSTM) is a novel DL approach (DL-LSTM) that has been used for different field applications like time series forecasting of wheat yield and productivity [21], irrigation requirement [22], predicting agricultural product sale volumes based on seasonal and historical data [23], and identification and classification of weeds [24]. Most of the image processing studies have used RGB images (or visible range imagery) to classify crop water stress [25,26]. Thermal imagery has been reported to be more robust for crop water stress characterization compared to RGB or multispectral imagery [27,28]. This is majorly due to the fact that the canopy emissivity can be highly sensitive to water content [8,9,29,30].

So far, crop water stress characterization has been carried out through traditional and destructive methods that often have restricted commercial applicability. Moreover, these techniques have been limitedly explored using robust computer-vision techniques (ML or DL models) for thermal infrared imagery inputs. Small unmanned aerial system (UAS)-based thermal and multispectral remote sensing is also being explored for high throughput crop water stress phenotyping. However, the frequency of data acquisition is limited to once or a few times a day and atmospheric interferences including weather conditions may severely impact the quality of thermal imaging. Additionally, onboard data processing potential for complex and robust algorithms is still limited for small UASs. Contrarily, proximal thermal imaging is subjected to the least atmospheric interference and imaging frequency constraints. These systems can continuously collect data at critical growth stages and also offer flexibility for custom modification to implement onboard edge processing algorithms for real-time decision support and management actuation. On the cost side, thermal and RGB imaging sensors and the UASs are still far more expensive than the proximal imaging systems, which can be custom-assembled using miniature sensing modules. However, such miniature sensing modules neither offer sufficient resolution nor desired image quality when integrated with UASs.

Obtaining robust data handling and the analytical pipeline is the major obstacle to deriving real-time decision support for crop management but is achievable using custom-assembled edge devices. This study is a step toward alleviating those obstacles and focuses on the evaluation of non-invasive and cost-effective thermal-RGB imaging with robust ML and DL models for stress characterization in winter wheat crops. This could be critical from a precision irrigation scheduling and management perspective and may potentially have high grower adaptability. Specific objectives for this two-year study were to (a) non-invasively assess the crop responses to two irrigation systems and four deficit irrigation treatments, and (b) identify the water-stressed and non-stressed crops by feature extraction using thermal-RGB imagery and function approximation approaches using crop physiological parameters and ambient weather inputs.

## 2. Materials and Methods

### 2.1. Experiment Design

**Figure 1:**
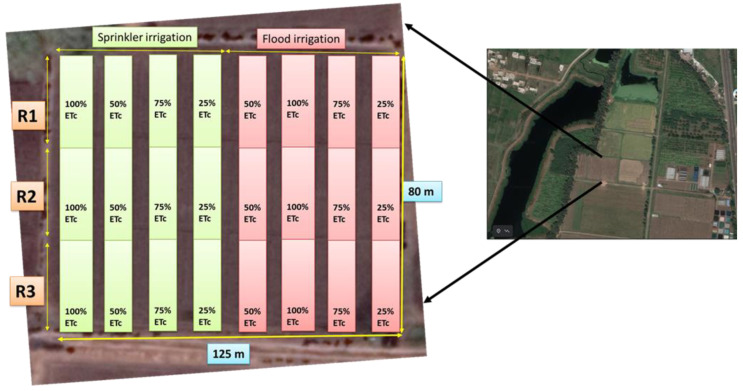
Experimental layout of the winter wheat crop irrigated at different rates using sprinkler and flood irrigation systems [32]. R—Replicates. Layout is prepared over google map.

Winter wheat (*Triticum aestivum* L., cv. HI 1544) was planted (November to April, 2019–2020, and 2020–2021) in the research farm (77.24° E, 23.18° N) of the Central Institute of Agricultural Engineering (CIAE), ICAR Bhopal, India (Figure 1). The meteorological data are being recorded at the institute observatory since 1985. According to Koppen’s classification (1934), Bhopal is a Mediterranean climatic zone with an average annual rainfall of about 1127 mm. The soil type is heavy clay (Vertisols) with clay content over 50% and moderate fertility with negligible salinity. Soil structure is sub-angular blocky with a field capacity of 29.5–32% (db) and wilting point of 18-19.5% (db). The average infiltration and percolation rates of the soil are 10–12 mm day^−1^ and 6.3–7.0%, respectively. The plots were irrigated using flood and sprinkler systems at four treatment rates: 100, 75, 50, and 25% of full crop evapotranspiration (ET_c_). Micro sprinklers of 120 lph discharge capacity (Make: Netafim) were installed at 3.5 m spacing. The reference crop evapotranspiration was calculated using weather data with the FAO56 Penman–Montieth method and standard non-stressed crop coefficient [31]. The seasonal ET_c_ of wheat during the first and second years of growth was 380 mm and 345 mm, respectively. The application efficiencies of 0.65 for flood irrigation and 0.90 for sprinkler irrigation were used as determined from the experiment trials on the same site using the measurements of water applied and the water retained in the crop root zone. A total of six irrigation cycles were implemented for 100% ET_c_ treatment (non-stressed) at sowing, crown root initiation (CRI), tillering, booting, flowering, and grain filling stages. One to three irrigation cycles were implemented for deficit treatments (75, 50, and 25% of ET_c_) at jointing, booting, and flowering stages.

### 2.2. Data Collection

RGB and thermal imagery were synchronously captured using a multifunctional custom integrated thermal-RGB imaging system. The system has a single board computer (B+, Raspberry Pi foundation), a thermal imaging module (8000–14,000 nm, HTPA, Heimann, Pixel resolution: 80 × 64, Horizontal and vertical FOV: 120° × 90°), an RGB imaging module (400–700 nm, Raspberry Pi V2, Sony IMX219, Raspberry Pi foundation, Pixel resolution: 3280 × 2464, HFOV: 62.2°, VFOV: 48.8°), a GPS receiver module (NEO 6M V2, Adafruit) for image geotagging, a capacitive touchscreen (LCD 800 × 400 mm, Robokit), a keypad (Robokits), and a power source (20,000 mAh, 5V/2A, MI power bank). The computer used the NOOBS operating system with module-pertinent libraries for different operations. Imagery data were collected at critical crop growth stages in the 2019 and 2020 growing seasons (five times in each season). Ground truth plant biophysical and soil parameters were also measured synchronously.

#### 2.2.1. Imagery Data

**Figure 2:**
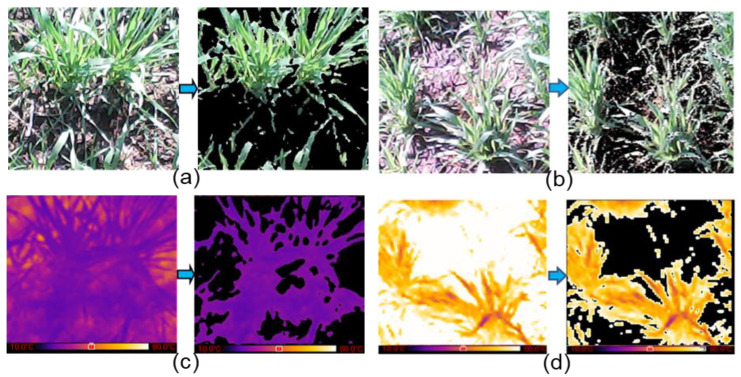
Sample raw and canopy masked RGB images ((**a**) non-stressed; (**b**) stressed) and thermal images ((**c**) non-stressed, (**d**) stressed) captured 35 days after sowing. Pseudo-color thermal images here are only for presentation and were scaled between 10 °C (RGB: [25, 25, 113]) and 80 °C (RGB: [235, 246, 255]).

The developed imaging system was placed 1 m from the crop and titled at 45° from the horizontal. A total of 3200 images (400 per treatment) were acquired (1600 RGB and 1600 thermal) in two seasons at Crown root initiation (20 days after sowing (DAS)), tillering (35 DAS) (Figure 2), jointing (70 DAS), flowering and milking (95 DAS), and dough (108 DAS) stages, between 11 am to 1 pm on clear sky days. Sample masked thermal and RGB canopy images for model training are shown in Figure 2. 

#### 2.2.2. Weather and Ground Truth Data

Weather data were acquired for the imaging days from a standard station (Indian Meteorological Department, Pune, India) installed at 300 m from the study site. The parameters included, pan evaporation (mm/day), rainfall (mm), maximum and minimum air temperature (°C), relative humidity (RH, %), and wind velocity (m/s). The ambient and soil ground truth data of air temperature (T_a_), RH, and soil moisture content (SMC) were collected for each treatment plot during the imaging campaigns each year. The T_a_ and RH parameters were recorded using the DHT22 module (Adafruit, New York, NY, USA). SMC was monitored in the root zone depth (0–150 mm typical to wheat crops grown in the experimental site, soil type: vertisols) using a soil moisture meter of 200 mm sensing probe length (ICT, MPM-160-B, Armidale, Australia). The probe was inserted at five different locations in each replication for measurement of soil moisture, acquiring 15 data points of soil moisture content per measurement. The relative water content (RWC) of leaves was calculated as the crop ground truth data [33]. For this, 10 matured and fully expanded leaves from each sample plot were collected and fresh weight was recorded on each sampling date, immediately following the imagery acquisition. Collected samples were then oven-dried at 70 °C, dry weight was recorded, and RWC was calculated. A total of 30 samples were collected per treatment per campaign amounting to a total of 150 samples per treatment in each year for RWC calculations. End-season yield was also recorded from 2 × 2 m areas from three plots in each replication, making nine sample points (36 m^2^ area) per treatment to characterize the effects of crop water stress.

#### 2.2.3. Statistical Analysis

The impact of irrigation type (flood and sprinkler), rate (100, 75, 50, and 25% ET_c_), and interaction of both on crop biophysical parameters were statistically evaluated using a one-way analysis of variance at a 5% level of significance [34].

### 2.3. Crop Water Stress Classification

Two different approaches (1) feature extraction-based (DL models: AlexNet, GoogLeNet, Inception V3, MobileNet V2, and ResNet50) and (2) function approximation-based ML models (Artificial neural network (ANN), K-nearest neighbors (KNN), Support vector machine (SVM), and Logistic regression (LR)); and a DL model (DL-LSTM) were adopted for crop water stress classification. Feature extraction-based models were trained on thermal as well as RGB imagery. Function approximation-based models were trained on ambient weather and soil parameters, and T_c_ inputs from thermal imagery.

Deep CNNs typically have complex architecture and some may require significant computational resources. All CNN model training and validation processes were performed on a desktop computer (Intel Core I7 Processor with base frequency 2.60 GHz, 16 GB RAM, 6 GB NVIDIA GeForce GTX 1660 Ti GPU) with Windows 10 operating system (64 bits). CNN models were developed in MATLAB 2019b using the deep learning and machine learning toolbox. All the models are detailed in the following sub-sections.

#### 2.3.1. Feature Extraction-Based Approaches

Five DL models were selected as the feature extraction-based approaches (1) AlexNet; (2) GoogLeNet; (3) Inception V3; (4) MobileNet V2; and (5) ResNet50. These models were selected for their extraordinary capabilities of automated feature extraction, easy and efficient training of the raw images with optimum computation resources, and their transferability to edge computation devices [10,17]. The selected models ranged from the simplest architecture (AlexNet and MobileNet V2) to the most complex architecture (GoogLeNet, Inception V3, and ResNet50) in order to evaluate their robustness and efficiencies for crop water stress prediction. Successful application of these models has been reported with accuracies up to 100% for crop abiotic and biotic stress classification in recent studies [35,36,37]. Standardized architectures were used in the models for performance comparisons (Table 1).

**Figure 3:**
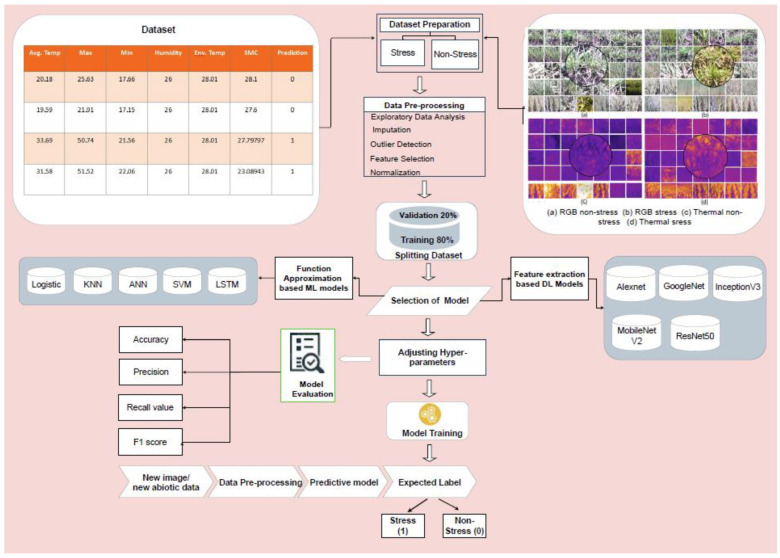
Data processing pipeline for stress prediction using selected deep learning and machine learning models.

The DL-based classification includes steps of pre-trained model selection, data pre-processing using morphological operators, data splitting, setting the training hyper-parameters, model training, model tuning, cross-validation, evaluation, and model testing (Figure 3). DL models were developed in MATLAB (version 2019a, Mathworks, Natick, Boston, MA, USA) using libraries of AlexNet, GoogLeNet, Inception V3, MobileNet V2, and ResNet50. The convolutional kernels in AlexNet were extracted using the cost function optimized by a stochastic gradient descent with momentum (Sgdm) algorithm. While GoogLeNet processes and classifies images by alternately factorizing the convolutions and regularization layers. To train a GoogLeNet model, the model’s loss 3-classifier, prob, and output layers were replaced by fully connected, softmax and output class layers which connected with other traditional layers. The inception V3 extracted (a) local features of the stressed crop by using small convolutions and (b) high abstracted features with large convolutions. The last three prediction layers in inception V3 were replaced by three new layers; fully connected, softmax, and classification output layer. These layers were interconnected with average pooling and a fully connected layer of the pre-trained DL. MobileNet V2 and ResNet50 are the very recently proposed DL models for classification problems. MobileNet V2 requires less computational power compared to conventional CNN. ResNet50 is a deep residual network that uses the shortcut connections by reducing the convolutional layers and by also solving the vanishing gradient issue typical to CNN. The residual modules in ResNet50 were used to connect different layers of CNN to improve the model performance [38]. 

Generalization can be poor for feature extraction-based models when the number of epochs and batch sizes are more than the optimum [39]. This is because the model can overlearn when trained on a specific dataset at large epochs and batch sizes, and may lose its performance and generalization capability when trained on new datasets. Conversely, the smaller epochs and bath sizes may lead to insufficient learning and underfitting of the model and hence may not perform as expected with the new datasets [40]. Therefore, to maximize the model performance and minimize their overfitting, optimum hyperparameter tuning is required. In this study, all the selected feature extraction-based models were extensively tuned with learning rates, solvers, epochs, and batch sizes as detailed in Table 2.

Collected thermal and RGB images (1600 each) were labeled into stressed and non-stressed classes by the domain experts based on the values of crop water stress indicators of SMC, RWC, and T_c_ (Table 3). After this, 80% of the labeled dataset (separately for thermal and RGB images) was used for DL model training based on features of object dimensions, pixel intensity, pixel values (T_c_), edges, etc. The remaining 20% of the labeled dataset was used for model validations and testing.

#### 2.3.2. Function Approximation-Based Approaches

Four ML models; ANN, KNN, LR, and SVM and a DL-based LSTM (DL-LSTM) were selected as the function approximation approaches for crop water stress classification. The ML models were selected as those provide an opportunity to analyze numerous features simultaneously unlike traditional methods. ANN is effective in learning complex nonlinear functions and segmenting data based on the learned weights. The input layer had four variables to extract features from 1600 samples while the output layer had one neuron to calculate the probability of each class [47]. KNN classifies a data point based on its distance from the maximum number of training data points in the neighborhood. Typically, KNN uses Euclidean, Minkowski, Manhattan, or Hamming distances out of which Minkowski distance has been reported to be more reliable [48] and was therefore selected in the model. LR classifies data points into discrete classes based on probability using a sigmoid or logistic function [49]. SVM shifts data points to a higher dimension using linear, non-linear, and radial kernels to achieve linear separability [50] and then identifies a hyperplane for the highest possible distance between data points of the two classes. DL-LSTM uses a chain of repeated modules comprising memory cells with a backpropagation algorithm to solve the classification problems. This model solves premature overfitting and vanishing gradient issues by using the previously stored information in the memory cell. The information is then used to generate the features during the training process to predict the output class [51]. ML models automatically tuned their hyperparameter values by using Bayesian optimization. The optimization minimizes the model loss based on the hyperparameter combination and yields the best possible set of parameters. Further, the models were trained and validated on these tuned hyperparameters. All function-approximation models were deployed on crop environment (T_a_, RH, and SMC) and temperature (T_c_, from thermal images) inputs for classification into stressed and non-stressed through binary outputs (0 or 1, Table 3). The models (operating parameters in Table 4) were developed in Python 3.7 with Keras and TensorFlow libraries.

### 2.4. Model Performance Evaluation

The performance of both feature extraction and function approximation-based models was evaluated through accuracy (A), sensitivity (S_e_), specificity (S_p_), precision (P), and F1 score parameters (Equations (1)–(5)). Accuracy is the correct prediction rate of non-stressed and stressed crops, precision is the fraction of true positive (T_S_) or correctly predicted stressed crop from an overall prediction of the stressed crop (P_S_), specificity is the true negative (T_N_) or correctly predicted non-stressed crops from the actual non-stressed crops (A_N_). Sensitivity represents a fraction of the correctly predicted stressed crops (T_S_) from the actual stressed crops (A_S_) and the F1 score is the harmonic mean of precision and sensitivity. The F1 score evaluates the accuracy of a binary classification problem as in this study, which aims to classify the crops into two classes (stressed and non-stressed). Often, the accuracy estimate is affected by true negatives and therefore F1 score is highly used over accuracy to seek a balance between the precision and recall (sensitivity) parameters and when there is an uneven class distribution (a large number of actual negatives).

(1) $$A=\frac{T_{S}+T_{N}}{T_{T}}$$

(2) $$S_{e}=\frac{T_{S}}{A_{S}}$$

(3) $$S_{p}=\frac{T_{N}}{A_{N}}$$

(4) $$P=\frac{T_{S}}{P_{S}}$$

(5) $$F1=\frac{2{*T}_{S}}{A_{S}+P_{S}}$$

where T_T_ is the total number of predictions. Stress/non-stress misclassification was represented by type1 (T_E1_) (false positive) and type2 errors (T_E2_) (false negative). T_E1_ is the number of actual stressed crops misclassified as non-stressed (row 1-column 2 of the confusion matrix) while T_E2_ is the number of actual non-stressed crops misclassified as stressed (row 2-column 1 of the confusion matrix).

## 3. Results

### 3.1. Plant Water Stress Indicators

**Figure 4:**
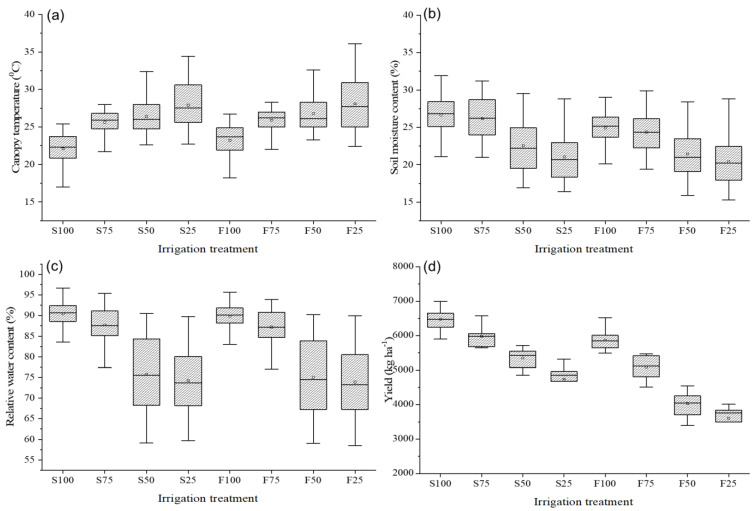
Variations in (**a**) canopy temperature; (**b**) soil moisture content; (**c**) relative water content; and (**d**) grain yield from wheat plots irrigated at different rates. S and F represent sprinkler and flood irrigations, respectively and the numbers followed by these letters denote irrigation rates levels as % of full crop evapotranspiration (ET_c_).

The thermal imagery derived canopy temperatures (T_c_ (°C)) under sprinkler irrigation at 100, 75, 50 and 25% of ET_c_ irrigation levels were 22.1 (2.0) (Mean, standard deviation (SD)), 25.6 (1.6), 26.4 (2.2), and 27.9 °C (3.0 °C), respectively (Figure 4). While T_c_ for flood irrigation at the above irrigation levels were 23.2 (2.0), 25.9 (1.5), 26.8 (2.4), and 28.1 °C (3.1 °C), respectively. Similarly, mean RWC (%) at selected sprinkler irrigation rates were 90.4 (2.7), 87.7 (4.2), 75.8 (9.4), and 74.2% (8.2%) while at corresponding irrigation levels in flood irrigation were 89.8 (2.7), 87.2 (4.3), 75.0 (9.4) and 73.9% (8.3%), respectively. The mean SMCs (%) for respective sprinkler irrigation were 26.6 (2.3), 26.2 (2.7), 22.5 (3.3), and 21.1% (3.0%) while those for respective flood irrigation were 24.9 (2.0), 24.4 (2.5), 21.4 (3.0), and 20.4% (3.1%). When analyzed statistically, T_c_, RWC, and SMC were significantly affected by the irrigation method (flood and sprinkler), irrigation rate (100, 75, 50, and 25% ET_c_), as well as their interaction (One-way ANOVA, *p* < 0.001). The RWC and SMC decreased with the decrease in irrigation level, while the T_c_ increased. Based on the categories detailed in Table 3, the mean T_c_ for the stressed crop was 26.6 °C (±2.6), and that for the non-stressed crop was 21.2 °C (±1.4). The mean RWC for the non-stressed crop was 92.2% (±1.5) and for the stressed crop was 78.9% (±9.2) while the mean SMC for the non-stressed crop was 27.1% (1.6) and for the stressed crop was 21.2% (±2.4).

### 3.2. Water Stress Prediction

#### 3.2.1. Feature Extraction-Based Approaches

**Figure 5:**
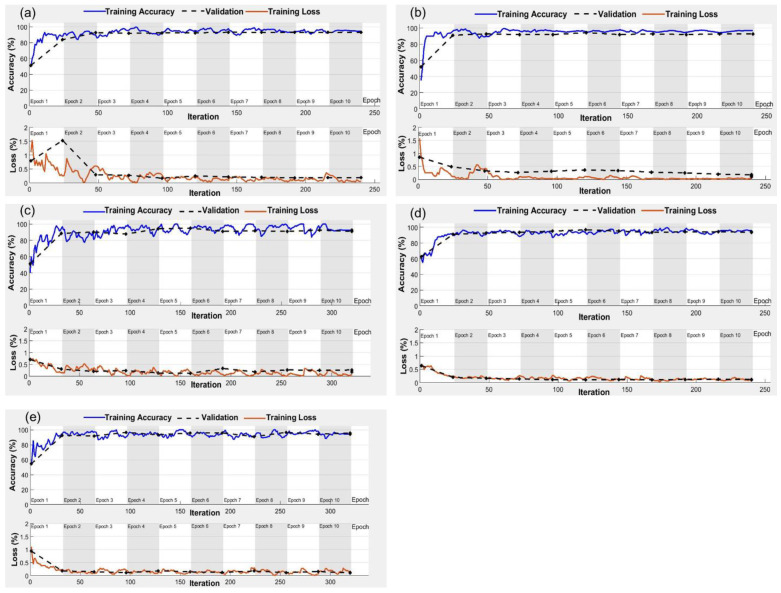
Accuracy and loss curves for (**a**) AlexNet; (**b**) GoogLeNet; (**c**) Inception V3; (**d**) MobileNet V2; and (**e**) ResNet50 models with RGB imagery inputs for crop water stress identification.

**Figure 6:**
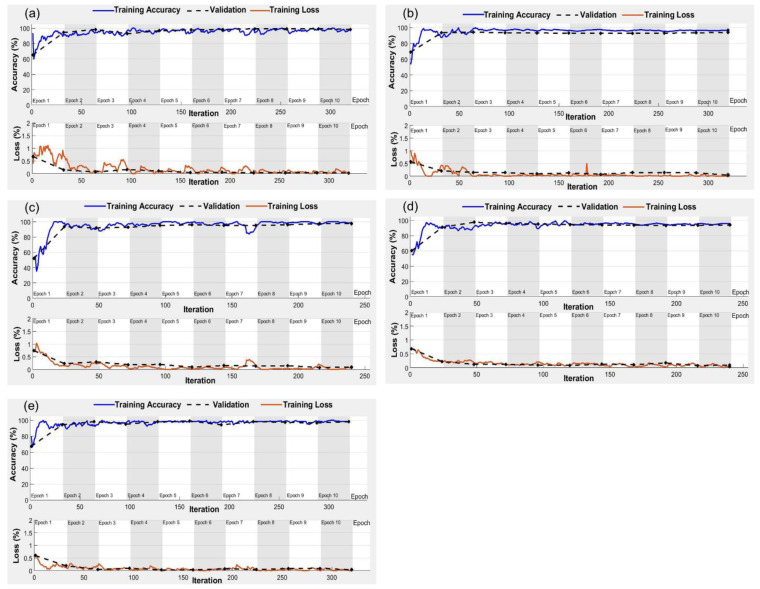
Accuracy and loss curves for (**a**) AlexNet; (**b**) GoogLeNet; (**c**) Inception V3; (**d**) MobileNet V2; and (**e**) ResNet50 models with thermal imagery inputs for crop water stress identification.

The performances of AlexNet, GoogLeNet, Inception V3, MobileNet V2, and ResNet50 models for RGB and thermal imagery were tested for different combinations of epochs and batch sizes (Table 5). The model training accuracies increased with the increase in the number of epochs from 5 to 10 and over-fitting was observed for all the models when epochs increased to 20. Over different epochs, accuracy increased with the increase in batch size from 5 to 20. For the batch size of 20 and 250 iterations, overfitting was observed in Inception and ResNet50 with RGB imagery inputs and in AlexNet, GoogLeNet, and ResNet50 with thermal imagery inputs (Table 5). Extensive hyperparameter tuning was performed with parameters listed in Table 2 to minimize overfitting and maximize the model accuracies. Post-tuning, the maximum training accuracies of 94.6%, 96.7%, and 95.6% were observed for AlexNet, GoogLeNet, and MobileNet V2, respectively with RGB imagery inputs at 10 epochs and batch size of 20 (Figure 5). While the Inception V3 and ResNet50 for RGB imagery inputs converged at 10 epochs, batch size of 15, and 300 iterations with respective accuracies of 92.7% and 97.1% (Figure 5c,e). For the thermal imagery inputs, the optimum hyperparameters were 10 epochs and a batch size of 15, which yielded maximum accuracies of 96.4%, 97.2%, and 98.5% for AlexNet, GoogLeNet, and ResNet50, respectively. Furthermore, 10 epochs and a batch size of 20 were found optimum for Inception V3 and MobileNet V2 models, and pertinent maximum accuracies were 98.0% and 95.3%, respectively (Figure 6). During hyperparameter tuning, the model overfitting reduced significantly at 10 epochs without sacrificing accuracy. The training accuracies fell below 50% for learning rates of 1 × 10^−4^ and 4 × 10^−4^ and went over 50% for the learning rate of 3 × 10^−4^. Moreover, the model overfitting was reduced when the solver was shifted from Sgdm to Adam. All the models converged with training accuracies > 90% at the learning rate of 3 × 10^−4^ and Adam as the solver (Figure 5 and Figure 6).

**Figure 7:**
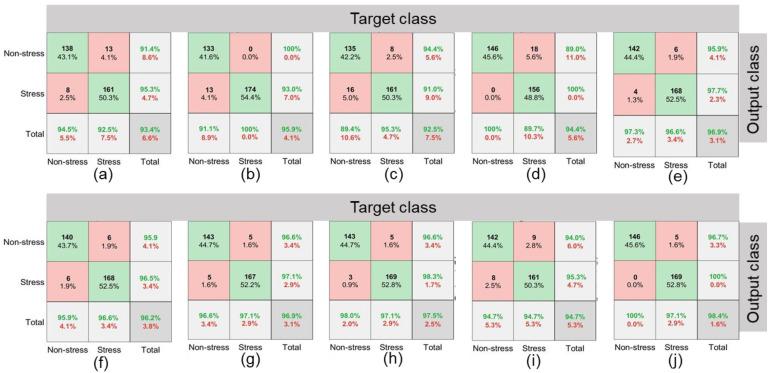
Confusion matrices for AlexNet, GoogLeNet, Inception V3, MobileNet V2, and ResNet50 models pertinent validation datasets of RGB imagery (**a**–**e**) and thermal imagery (**f**–**j**). Cell values (%) in row 1 column 2 represent type 1 error (T_E1_) while those in row 2 column 1 represent type 2 error (T_E2_) (details in Section 2.4). Numbers (% and actual counts) in green color indicate prediction accuracy while those in red color are prediction errors for stressed and non-stressed crop classes. Numbers in green box represent correct prediction and those in red box represent misclassification of non-stressed/stressed classes.

The training time elapsed for AlexNet, GoogLeNet, Inception V3, MobileNet V2, and ResNet50 was 76, 92, 609, 149, and 217 min with RGB imagery inputs, and 42, 88, 287, 134, 168 min with thermal imagery inputs, respectively. While the classification of an independent image using trained models into stressed/non-stressed class consumed less than 5 s. The overall validation accuracies (combined for stressed and non-stressed classes) for AlexNet, GoogLeNet, Inception V3, MobileNet V2, and ResNet50 models were 93.4%, 95.9%, 92.5%, 94.4%, and 96.9%, respectively with RGB imagery inputs (Figure 7). The highest precision (100%) and F1 score (96.6%) were observed for GoogLeNet and ResNet50, respectively while maximum sensitivity was achieved for MobileNet V2 (Table 6). Pertinent to thermal imagery inputs, overall validation accuracies (combined for stressed and non-stressed classes) with AlexNet, GoogLeNet, Inception V3, MobileNet V2, and ResNet50 models were 96.2%, 96.9%, 97.5%, 94.7%, and 98.4%, respectively. Alike RGB imagery, ResNet50 for thermal imagery had the highest precision (96.7%), sensitivity (100%), and F1 score (98.3%) (Table 6). Additionally, the accuracies were higher for the models with thermal imagery inputs compared to those with RGB imagery inputs. The individual accuracy and errors for all the feature extraction-based models with validation datasets are shown in Figure 7. The mean errors were higher for the RGB imagery compared to the thermal imagery irrespective of the selected models. 

#### 3.2.2. Function Approximation-Based Approaches

**Figure 8:**
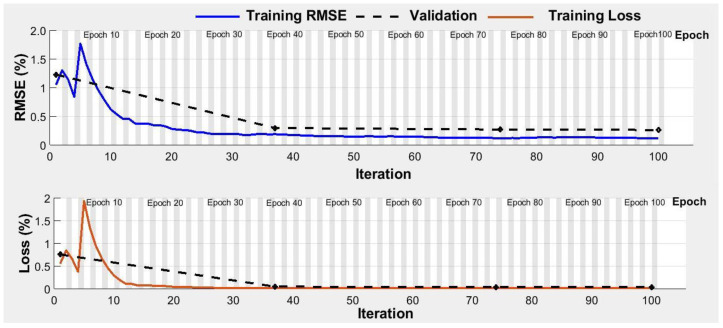
Accuracy and loss corves for Long Short Term memory based deep learning model for crop water stress identification.

**Figure 9:**
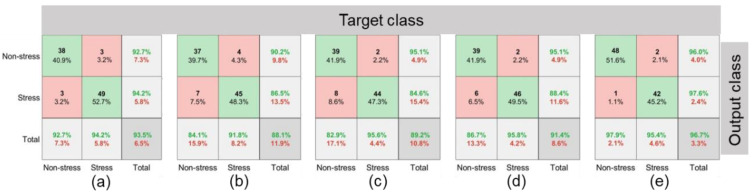
The confusion matrices for function approximation-based (**a**) ANN; (**b**) KNN; (**c**) LR; (**d**) SVM; and (**e**) DL-LSTM models. Cell values (%) in row 1 column 2 represent type 1 error (T_E1_) while those in row 2 column 1 represent type 2 error (T_E2_) (details in Section 2.4). Numbers (% and actual counts) in green color indicate prediction accuracy while those in red color are prediction errors for stressed and non-stressed crop classes. Numbers in green box represent correct prediction and those in red box represent misclassification of non-stressed/stressed classes.

Amongst the function approximation approaches, the highest prediction accuracy was obtained with the DL-LSTM model (96.7%) followed by ANN (93.5%), SVM (91.4%), LR (89.2%), and KNN models (88.1%) (Table 6). Moreover, the precision, sensitivity, and F1 score were also highest for the DL-LSTM (96.0, 97.9, and 97.0%, respectively) compared to other ML models. The training and validation accuracies with DL-LSTM showed early convergence for which the loss on the validation dataset reached minima at 40 epochs (Figure 8). The T_E1_ for ANN, KNN, LR, SVM, and LSTM were 3.2, 4.3, 2.2, 2.2, and 2.1%, respectively and T_E2_ were 3.2, 7.5, 8.6, 6.5, and 1.1%, respectively (Figure 9). The DL-LSTM outperformed ML models with the lowest mean error (Figure 9).

## 4. Discussion

Sprinkler irrigation applies a predetermined quantity of water and wets the entire canopy, unlike traditional flood irrigation. This cools down the microclimate and increases relative air humidity to reduce the microclimate’s water demand [52]. This could be the reason for lower T_c_ in all the sprinkler irrigation treatments compared to the corresponding flood irrigation treatments (Figure 4). Lowered microclimate water demand could have also resulted in lower soil moisture depletion from the root zone and therefore higher SMC with sprinkler irrigation [53]. In addition to sufficient SMC, sprinkler irrigation results in lower deep percolation and nutrient leaching compared to conventional flood irrigation [54,55,56]. This could have resulted in a higher average yield for sprinkler irrigation treatment plots (5719 kg/ha) compared to the flood irrigation treatment plots (4898 kg/ha). With the projected future climate change impacts in the form of low rainfall frequencies and high ambient temperatures, crop water stresses are further expected to multiply, which will multiply the penalties in yield potentials [3]. Therefore, stress-tolerant crop cultivars need to be developed and planted for uncompromised yield goals. As also reported in our prior work based on canopy reflectance [57], water stress started to occur before the CRI stage in both methods of irrigation. RWCs were lower at late jointing and flowering stages in case of flood irrigation. Water stress at the flowering stage can result in significant yield and biomass reductions [58] suggesting that it is also influenced by the phenological growth stage. For robust analysis of this aspect, large datasets are being collected at each phenological growth stage. Water stress lowers CO_2_ availability due to stomatal closure, thereby affecting photosynthesis and ultimately growth, yield, and biomass [59,60].

CNNs have been increasingly used for plant phenotyping applications over the past decade for their capability of modeling complicated plant processes by distinguishing and extracting regularized data patterns [61,62]. It is for this reason; CNN models were highly accurate in predicting stressed and non-stressed crops using thermal and RGB imagery. Chlorophyll is vital for photosynthesis, while carotenoids are critical non-enzymatic antioxidants. Water stress reduces chlorophyll and carotenoid contents, as well as the ratio of chlorophyll ‘a’ to ‘b’, leading to leaf coloration changes. This is the reason for RGB images also yielding satisfactory accuracy of up to 94.6% for tracing leaf color changes [63]. Compared to RGB imagery, thermal imagery is a more detailed and better presenter of the crop stress that alters the emissivity patterns proportionally [64,65]. The canopy temperature is affected by the microclimate conditions and the available soil moisture [53]. This is the reason for the relatively lower accuracy of water stress detection with RGB images (94.6%) than with thermal images (96.7%) irrespective of the selected DL models (Table 5). A similar observation is reported in a prior study [64] where higher accuracy was obtained with thermal imagery (89%) compared to RGB imagery (82%) for wheat ear counting using DCNN models. Since thermal imaging is often affected by the wind or RH factors of the environment, the quality of data will be critical for training the DL models, especially when acquired using aerial platforms [30,64]. Therefore, to maintain thermal data quality, imaging campaigns were launched when wind velocities were below 5 kmph. The ResNet50 had relatively the highest accuracy among the feature extraction models. Although it is the basic version of GoogLeNet and Inception V3, the performance would be highly impacted by the quality of input imagery, size, and robustness of the dataset, especially for the agricultural environments. ResNet50 addresses the neural network degradation problem by introducing identity mapping, which results in the disappearance of gradient parameters and the non-ideal convergence effect on the deeper networks [66,67]. This feature contributed to the enhanced performance of ResNet50 compared to the other models thereby suggesting the suitability of ResNet50 for agricultural applications for various crop biotic and abiotic stress characterizations. CNN models were also applied to thermal imagery for water stress classification in maize under well-irrigated, moderately irrigated, and water-stressed treatments, obtaining an overall accuracy of 89% [68]. Color and grey images of maize were also used as inputs to the DCNN model for water stress identification where stress identification and classification accuracies were 98% and 95%, respectively [26]. The inception-ResNet V2 framework utilized for water stress identification in sugarcane yielded an accuracy of 83% with available soil water capacity as input [65]. Thus far, most of the computer vision models have utilized single-dimensional data inputs, unlike this study which advances water-stress identification in wheat crops using multidimensional data inputs. Multidimensional data modeling enhances the robustness and applicability of developed approaches across various agroclimatic conditions.

Crop growth or its water stress response is not necessarily linear to the weather or soil conditions [69]. Therefore, the linear (LR) and non-linear function approximation approaches (ANN, KNN, SVM, and DL-LSTM) were evaluated to predict the stress class of the crop. ANN and SVM had a better stress prediction accuracy (Table 6) compared to KNN and LR possibly due to two reasons (1) KNN or LR either use locally linear segments or a generalized linear approach for making predictions [66,69] and (2) KNN and LR models train on an unsupervised learning approach, unlike ANN and SVM, which train on a supervised learning approach [18]. ANN and SVM had comparable accuracies for crop stress prediction. However, SVM suits small datasets; while ANN can process relatively larger datasets. Therefore, ANN would have more confidence in prediction classes.

Crop phenotyping with traditional function approximation approaches (ML models) is often subjective compared to the advanced DL-LSTM approach as those require manual feature selection of T_c_, T_a_, RH, and SMC. This restricts the robustness and accuracy of the ML models. Therefore, DL-LSTM outperformed the traditional ML models (Figure 9) due to its automated and stabilized feature selection advantage [12,70]. This was supported by minimum model loss compared to other function approximation-based ML models. DL-LSTM not only integrates the thermal imagery features employed in DL models but also combines the auxiliary soil and weather data inputs, of function approximation models. This eventually led to its superior performance over the other ML models evaluated in this study as well as in prior studies of crop stress and yield phenotyping [51] or irrigation forecasting [22]. However, GoogleNet, Inception V3, and ResNet50 provided comparable or higher stress prediction accuracy compared to the DL-LSTM model (Table 6). Stress/non-stress misclassification could be minimized through improved data sampling, increasing training data size, and optimizing hyper-parameters, or by merging different ML and DL models for crop’s thermal emissivity and environment data inputs. Among the feature extraction and function approximation-based approaches, the feature extraction-based models outperformed all the function approximation-based models for water stress classification.

The CNN models evaluated in this study can be adopted for water stress identification in other wheat cultivars while for other crops and their cultivars, sufficient data acquisition, model training, and validations would be required. Along similar lines, gathering sufficient data at different crop phenological stages will enable growth stage-wise accuracy evaluation of ML and DL models in future studies. The developed algorithms required below 5 sec to be successfully implemented on independent images for classification into stressed/non-stressed classes. This is critical from a real-time stress diagnosis and management perspective. Trained algorithms are therefore transferrable to handheld or edge devices for real-time stress detection by breeders, researchers, farmers, and students, among others. For commercial adoption of the developed and tested approaches, capital investment would be initially required following which high returns may be expected through improvements in crop stress mitigation and management at reduced costs [11].

## 5. Conclusions

The canopy temperatures, relative water content, soil moisture content, and grain yield for the wheat crop were significantly affected by the irrigation type and rates. Lower T_c_ and higher RWC, SMC, and yield were observed for irrigation at 100% of ET_c_ compared to deficit irrigation (75, 50, and 25% of ET_c_). Moreover, a comparable or higher yield was observed for sprinkler irrigation compared to conventional flood irrigation and amounted to 20% of the water savings.

Thermal images resulted in higher crop water stress classification accuracy (94.7–98.4%) compared to RGB imagery (92.5–96.9%). Moreover, the DL models (including DL-LSTM) performed better than the ML models for stressed and non-stressed crop classification. Among the function approximation-based approaches, DL-LSTM had the highest accuracy (96.7%). Among the feature extraction-based methods, ResNet50 had the highest accuracy of 96.9% and 98.4% with RGB and thermal imagery inputs, respectively.

Overall, DL models with thermal imagery inputs could be highly efficient for crop water stress phenotyping. As a future scope, feature extraction-based DL models could be implemented on edge-computing devices for real-time water stress monitoring and actuation of irrigation systems through the internet of things.

## Figures and Tables

**Table 1:** Architecture parameters in the feature extraction-based models for crop water stress classification.

Architecture Parameters	AlexNet	GoogLeNet	Inception V3	MobileNet V2	ResNet50
Input image size	227 × 227 × 3	224 × 224 × 3	299 × 299 × 3	224 × 224 × 3	224 × 224 × 3
No. of layers	25	141	316	154	177
Relu layer	7	57	95	35	49
Max Pooling layer	3	13	4	-	01
Convolutional layers	5	57	94	35	53
Dropout layer	2	1	-	-	-
Fully connected	3	1	1	1	1
Fully connected layer Function	FC8	Loss3 classifier	Predictions	Logits	FC1000
Depth	8	22	48	53	50
Parameters	61 × 10^6^	7 × 10^6^	23.9 × 10^6^	3.5 × 10^6^	25.6 × 10^6^

**Table 2:** Hyperparameter tuning considerations to reduce overfitting and performance enhancement of the feature extraction-based models.

Parameters	Value
Epoch	5, 10, and 20
Batchsize	5, 10, 15, and 20
Iterations	250 and 300
Solver	Sgdm and Adam
Learning rate	1 × 10^−4^, 2 × 10^−4^, and 3 × 10^−4^

Sgdm: stochastic gradient descent with momentum; Adam: adaptive moment estimation.

**Table 3:** Crop and auxiliary data ranges for stressed and non-stressed labeling.

Crop Label	Parameter	Output	References
Stressed	Canopy temperature (T_c_): >23 °C & Relative water content (RWC): <90% & Soil moisture content (SMC): <25%	0	[41,42,43,44,45,46]
Non-Stressed	neither of the “stressed” conditions	1	

**Table 4:** Training parameters of function approximation-based classification models.

Function Approximation Model	Parameters
Artificial Neural Network (ANN)	Hidden layers: 2 Neurons: 64, 32 Learning rate (alpha): 0.01 Activation functions: sigmoid Batch size: 8 Number of epochs: 300 Optimizer: Adam Loss function: binary cross entropy
Kernel Nearest Neighbour (KNN)	Number of Neighbors (K): 8 Distance Metric: minkowski Weights: uniform Algorithm: ball-tree
Logistic Regression (LR)	Penalty parameter: L1 Inverse of regularization parameter (C): 5 Maximum iteration: 100 Tolerance: 0.0001
Support Vector Machine (SVM)	Kernel Type (Kernel): RBF (Radial basis Function) Penalty parameter (C): 100 bandwidth parameter (gamma): 0.001 Degree of the polynomial kernel: 3
Deep Learning-Long Short Term Memory (DL-LSTM)	Number of neurons: 180 Epochs: 200 Batch size: 10 Optimizer: Adam Number of hidden layers: 2 Loss activation function: MAE (Mean absolute error)

Adam: adaptive moment estimation.

**Table 5:** Training accuracies of feature extraction-based models to characterize wheat water stress using RGB and thermal imagery inputs under different epoch and batch size combinations.

		Accuracy (%)				
Epochs	Batch Size	AlexNet	GoogLeNet	Inception V3	MobileNet V2	ResNet50
Feature Extraction-Based Approaches with RGB Imagery Inputs						
5	5	90.4	95.2	91.9	95.1	96.3
5	10	89.4	94.3	90.5	93.0	92.7
5	15	92.3	94.6	90.4	92.3	93.5
5	20	92.6	95.0	90.8	92.7	94.6
10	5	93.8	95.5	92.2	93.1	95.8
10	10	92.7	95.7	92.4	94.2	95.1
10	15	93.4	95.9	92.7	94.4	97.1
10	20	94.6	96.7	93.6	95.6	97.2
20	5	95.3	97.2	93.8	94.4	92.3
20	10	95.6	97.5	94.2	95.5	97.2
20	15	96.6 *	98.0	94.5	96.7 *	97.9 *
20	20	96.2	98.2 *	95.0 *	96.1	95.8
Feature extraction-based approaches with thermal imagery inputs						
5	5	94.4	96.2	97.0	94.8	95.9
5	10	95.9	95.8	96.8	94.7	95.9
5	15	96.4	95.4	95.9	93.1	98.4
5	20	92.7	96.0	96.2	92.7	97.6
10	5	94.5	96.5	97.2	93.5	96.7
10	10	96.0	96.7	97.4	94.2	97.3
10	15	96.4	97.2	97.5	94.7	98.5
10	20	96.5	97.2	98.0	95.3	98.7
20	5	97.2	97.6	98.2	97.2	99.0
20	10	97.4	98.1	98.5	97.5	99.2
20	15	98.2 *	98.5 *	98.7 *	98.1 *	99.5 *
20	20	98.0	98.0	98.5	97.9	99.0

* Highest accuracy for the epoch and batch size combinations.

**Table 6:** Validation performance of feature extraction and function approximation models to characterize wheat water stress.

Models	Accuracy (%)	Precision (%)	Sensitivity (%)	F1 Score (%)
Feature Extraction-Based Approaches with only RGB Imagery Inputs				
AlexNet	93.4	91.4	94.5	92.2
GoogLeNet	95.9	100	91.1	95.3
Inception V3	92.5	94.4	89.4	91.8
MobileNet V2	94.4	89.0	100.0	94.1
ResNet50	96.9	95.9	97.3	96.6
Feature extraction-based approaches with only thermal imagery inputs				
AlexNet	96.2	95.9	95.9	95.9
GoogLeNet	96.9	96.6	96.6	96.6
Inception V3	97.5	96.6	98.0	97.3
MobileNet V2	94.7	94.0	94.7	94.3
ResNet50	98.4	96.7	100.0	98.3
Function approximation-based approaches (with RWC, SMC, Tc, and RH inputs)				
ANN	93.5	92.7	92.7	93.0
KNN	88.1	90.2	84.1	86.9
LR	89.2	95.1	82.9	88.6
SVM	91.4	95.1	86.7	90.8
DL-LSTM	96.7	96.0	97.9	97.0

## Data Availability

Data will be made available on personalized requests due to restrictions from the parent organization.

## Abbreviations

**Abbreviation**	**Expanded Form**
Adam	Adaptive Moment Estimation
AN	Actual Non-Stressed Crop
ANN	Artificial Neural Network
ANOVA	Analysis of Variance
CIAE	Central Institute of Agricultural Engineering
CNN	Convolution Neural Network
DAS	Day After Sowing
DCNN	Deep Convolution Neural Network
DL	Deep Learning
DL-LSTM	Deep Learning-Long Short Term Memory
ET_c_	Evapotranspiration
F1	F1 Score
ICAR	Indian Council of Agricultural Research
KNN	Kernel Nearest Neighbor
LR	Logistic Regression
LSTM	Long Short Term Memory
MAE	Mean Absolute Error
ML	Machine Learning
P	Precision
PS	Correctly Predicted Stressed Crop From all the predictions
RBF	Radial Basis Function
RF	Random Forest
RGB	Red Green Blue
RH	Relative Humidity
RWC	Relative Water Content
SD	Standard Deviation
S_e_	Sensitivity
Sgdm	Stochastic Gradient Descent with Momentum
SMC	Soil Moisture Content
S	Specificity
VM	Support Vector Machine
T_a_	Air Temperature
T_c_	Canopy Temperature
T_E1_	Type 1 Error
T_E2_	Type 2 Error
TN	True Negative
TS	True Positive
UAS	Unmanned Aerial System
